# Disorder-specific cingulo-opercular network hyperconnectivity in pediatric OCD relative to pediatric anxiety

**DOI:** 10.1017/S0033291721003044

**Published:** 2023-03

**Authors:** Hannah C. Becker, Luke J. Norman, Huan Yang, Christopher S. Monk, K. Luan Phan, Stephan F. Taylor, Yanni Liu, Kristin Mannella, Kate D. Fitzgerald

**Affiliations:** 1 Department of Psychology, University of Michigan, Ann Arbor, MI, USA; 2 Department of Psychiatry, University of Michigan, Ann Arbor, MI, USA; 3 National Human Genome Research Institute, Bethesda, MD, USA; 4 The Second Xiangya Hospital, Central South University, Changsha, China; 5 Department of Psychiatry and Behavioral Health, The Ohio State University, Columbus, OH, USA

**Keywords:** Brain connectivity, cingulo-opercular network, OCD, orbito–striatal–thalamic network, pediatric anxiety

## Abstract

**Background:**

Prior investigation of adult patients with obsessive compulsive disorder (OCD) has found greater functional connectivity within orbitofrontal–striatal–thalamic (OST) circuitry, as well as altered connectivity within and between large-scale brain networks such as the cingulo-opercular network (CON) and default mode network (DMN), relative to controls. However, as adult OCD patients often have high rates of co-morbid anxiety and long durations of illness, little is known about the functional connectivity of these networks in relation to OCD specifically, or in young patients near illness onset.

**Methods:**

In this study, unmedicated female patients with OCD (ages 8–21 years, *n* = 23) were compared to age-matched female patients with anxiety disorders (*n* = 26), and healthy female youth (*n* = 44). Resting-state functional connectivity was used to determine the strength of functional connectivity within and between OST, CON, and DMN.

**Results:**

Functional connectivity within the CON was significantly greater in the OCD group as compared to the anxiety and healthy control groups. Additionally, the OCD group displayed greater functional connectivity between OST and CON compared to the other two groups, which did not differ significantly from each other.

**Conclusions:**

Our findings indicate that previously noted network connectivity differences in pediatric patients with OCD were likely not attributable to co-morbid anxiety disorders. Moreover, these results suggest that specific patterns of hyperconnectivity within CON and between CON and OST circuitry may characterize OCD relative to non-OCD anxiety disorders in youth. This study improves understanding of network dysfunction underlying pediatric OCD as compared to pediatric anxiety.

## Introduction

Obsessive compulsive disorder (OCD) is a debilitating condition that affects 2–3% of the U.S. population (Ruscio, Stein, Chiu, & Kessler, 2010) through recurring and unwanted thoughts (obsessions) and the repetitive behaviors aimed at lessening obsession-associated anxiety [compulsions; American Psychiatric Association (APA), 2013]. In addition to OCD-specific symptoms, 50–75% of adult U.S. OCD patients also suffer from co-morbid anxiety and over 60% experience a mood disorder (Janowitz et al., 2009; Ruscio et al., 2010). In pediatric OCD, mood disorders are less common (Brakoulias et al., 2017; Ivarsson, Melin, & Wallin, 2008); however, co-occurring anxiety symptoms are prevalent, with 45–60% of pediatric OCD patients also meeting criteria for one or more anxiety disorders (Ivarsson et al., 2008; Tanidir et al., 2015). This comorbidity complicates the separation of OCD-specific pathology from non-OCD anxiety pathology, even in affected children who are near illness onset and less likely than adults to have developed additional psychopathology (e.g. depression) or medication exposures that may impact neural circuitry underlying OCD. Gender differences have also been observed in OCD. OCD tends to be more common in boys than girls in childhood, but in adolescence and adulthood, the reverse pattern is observed (Mathes, Morabito, & Schmidt, 2019). Differences in the symptom dimensions, symptom severity, and co-morbidities experienced between males and females with OCD have also been identified (Mathes et al., 2019). For example, higher rates of some anxiety disorders in female than male youth with OCD have been reported (Masi et al., 2010), consistent with greater prevalence and severity of anxiety disorders in females than males in the general population (Beesdo, Knappe, & Pine, 2009).

Previous models of the pathophysiology of OCD have focused on dysfunction within the corticostriatal circuits of the brain. These circuits are anatomically segregated loops involved in goal-directed behavior, that pass through the cortex, the striatum, the thalamus, and then back to cortex (Alexander, DeLong, & Strick, 1986; Haber, 2016). Using neuroimaging tools such as functional magnetic resonance imaging (fMRI) and positron-emission tomography, researchers have found that corticostriatal system activity in patients with OCD increases during symptom provocation and resolves after effective treatment (Menzies et al., 2008; Nakao, Okada, & Kanba, 2014; Saxena & Rauch, 2000). Within this corticostriatal system, in contrast to healthy individuals, OCD is most consistently associated with dysregulation in orbitofrontal cortex (OFC), dorsal striatal, and thalamic regions of an orbitofrontal–striatal–thalamic (OST) circuit in both children and adults (Fitzgerald et al., 2011; Kodaira et al., 2012; Norman et al., 2019; Rosenberg & Keshavan, 1998). In addition to OST hyperactivity during symptom provocation (Banca et al., 2015; Breiter et al., 1996; Brennan et al., 2015), compared to healthy controls (HCs), adults with OCD exhibit OST hyperactivity during habit-responding, but hypoactivity during inhibitory control and reward tasks (Kang et al., 2013; Norman et al., 2018, 2019). Prior resting-state fMRI research has revealed hyperconnectivity of regions in the OST circuit, suggestive of excess signaling in adults and children with OCD compared to HCs (Apergis-Schoute et al., 2018; Fitzgerald et al., 2011; Harrison et al., 2009; Hou et al., 2014; Jang et al., 2010; Sakai et al., 2011).

Extending beyond the study of corticostriatal circuitry, neuroimaging research of OCD has examined potential differences in the behavior of large-scale intrinsic functional brain networks (Peterson, Thome, Frewen, & Lanius, 2014; Posner et al., 2017; Stern, Fitzgerald, Welsh, Abelson, & Taylor, 2012; Tian et al., 2016), such as the cingulo-opercular network (CON) and the default mode network (DMN). Intrinsic networks such as the CON and DMN can be identified with resting-state fMRI as distributed regions that display highly correlated patterns of a low-frequency blood-oxygen-level-dependent signal. These correlations are thought to index functional connectivity between regions that are anatomically connected, as demonstrated by tract-tracing studies in non-human primates (Carmichael & Price, 1996; Goldman-Rakic, 1988; Haber, 2003; Mesulam, 1990). The CON encompasses posterior medial frontal cortex (pMFC) and fronto-operculum (Norman et al., 2019; Power et al., 2011; Sestieri, Corbetta, Spadone, Romani, & Shulman, 2014) and is engaged when directing attention externally to salient events in the environment (Dosenbach et al., 2006; Neta, Schlaggar, & Petersen, 2014). A meta-analysis that included studies of both children and adults found CON hyperactivity during error-processing in addition to CON hypoactivity during inhibitory control in patients with OCD as compared to HCs. These findings implicate the CON in cognitive processes that may prevent patients from stopping unwanted thoughts or behaviors that occur (Norman et al., 2019). Previous studies have shown an altered relationship within and between the CON and other brain networks at rest in OCD patients in contrast to HCs (Fan et al., 2017; Tomiyama et al., 2019), particularly the DMN (Beucke et al., 2014), and including in pediatric patients (Fitzgerald et al., 2010).

The DMN is comprised of nodes in posterior cingulate cortex (PCC), precuneus, ventral medial prefrontal cortex (vmPFC), angular gyrus (AG), and medial temporal cortex (Andrews-Hanna, Reidler, Huang, & Buckner, 2010; Fransson & Marrelec, 2008; Greicius, Krasnow, Reiss, & Menon, 2003). The DMN deactivates during external cognitively demanding tasks (Gusnard & Raichle, 2001; Shulman et al., 1997), and is inversely correlated with the CON (Fox et al., 2005). Relative to healthy individuals, in pediatric and adult patients with OCD, regions of the DMN have been shown to deactivate to a lesser degree during externally oriented tasks (Brennan et al., 2015; Stern et al., 2011, 2012), to have reduced connectivity with each other (Fitzgerald et al., 2010; Peng et al., 2014), and in some instances, display greater connectivity with other large-scale networks, including the CON (Beucke et al., 2014; Harrison et al., 2013; Hou et al., 2013; Posner et al., 2017; Yun et al., 2020). Since the DMN is engaged during introspective, self-referential processing, prior research has noted the potential role of the DMN in generating repetitive patterns of thinking associated with OCD (Stern et al., 2012).

Although earlier research provides strong support for the involvement of OST, CON, and DMN circuits in OCD, several methodological limitations hinder conclusions about the specificity of findings. For example, OCD patients in these studies are often taking selective serotonin reuptake inhibitors (e.g. Apergis-Schoute et al., 2018; Beucke et al., 2014; Fitzgerald et al., 2010, 2011), which have been found to change OST connectivity in youth with OCD (Bernstein et al., 2019). Furthermore, most studies of unmedicated OCD compared to HC groups include adult patients, who have likely experienced OCD symptoms for many years at the time of the study (Chen et al., 2018; Hou et al., 2013; Posner et al., 2014; Sakai et al., 2011). Consequently, the relationship between these large-scale brain networks at the early-stages of OCD onset, without the effects of compensatory neural response or pharmacological treatment, is not well understood. Furthermore, as prior research has historically sampled OCD patients with high rates of co-morbid anxiety disorders (Beucke et al., 2014; Chen et al., 2018; Cocchi et al., 2012; Fitzgerald et al., 2013; Harrison et al., 2013; Hou et al., 2012; Jang et al., 2010; Jung et al., 2017; Sylvester et al., 2012), it remains unclear to what extent the differences in within- and between-network connectivity in ‘OCD groups’ are specific to OCD. However, studies that examined resting-state network connectivity in adults with OCD and non-OCD anxiety independently have found dysfunctional connectivity in both the DMN and the CON as compared to HCs (Beucke et al., 2014; Peterson et al., 2014; Rabany et al., 2017; Xu et al., 2019). However, few studies have directly examined resting-state connectivity of the OST, CON, or DMN in anxiety disorders without OCD (Peterson et al., 2014; Sylvester et al., 2012), leaving the alterations of large-scale intrinsic networks that may differentiate between these disorders relatively unexplored.

The current study builds upon prior neuroimaging research showing evidence for dysfunction in OST, CON, and DMN in pediatric OCD. Our predictions were informed by a large body of research that supports alterations of OST circuitry in OCD, in contrast to relatively minimal evidence implicating this circuitry in non-OCD anxiety disorders (Peterson et al., 2014; Sylvester et al., 2012). Specifically, we hypothesized that there would be hyperconnectivity within the OST circuit in the OCD group compared to the other two groups and that any differences in connectivity strength between the OST network and the CON or DMN would be unique to OCD patients. In contrast, we hypothesized that differences in within-network DMN or CON connectivity, as well as between-network CON to DMN connectivity, would be shared by both the OCD and anxiety control (AC) groups, consistent with previously published theoretical models and empirical research (de Vries et al., 2019; Fitzgerald et al., 2010; Shanmugan et al., 2016; Sylvester et al., 2012). Based on this research, we expected to see hyperconnectivity within the CON and between the CON and DMN in both the OCD and AC groups as compared to the HC group.

## Methods

### Participants

Ninety-seven unmedicated females aged 8–21 years with a primary diagnosis of OCD (*n* = 25), one or more non-OCD anxiety disorders (*n* = 27), or no diagnosis (*n* = 45) underwent resting-state fMRI at the University of Michigan Medical School (UMMS). Early-onset OCD more commonly affects young boys whereas later-onset OCD and non-OCD anxiety disorders are more common in girls (Mathis et al., 2011; Merikangas et al., 2010); thus, the inclusion of only girls eliminated potential confounding between groups by gender representation and age. Primary diagnosis determined group membership and was defined by the primary source of impairment at study entry, based on structured clinical interview using the Kiddie Schedule for Affective Disorders and Schizophrenia, or the Structured Clinical Interview for DSM-V if the subject was 18 years or older. Secondary (i.e. lower severity) anxiety disorders were included in the OCD group. By contrast, no OCD diagnoses were present among patients in the AC group. For generalizability, patients with co-morbid tic, anxiety, dysthymia, or depression not otherwise specified were included as long as OCD (in the OCD group), or a non-OCD anxiety disorder was the primary source of impairment. Major depressive disorder was absent from the OCD group but occurred as a secondary diagnosis (i.e. less impairing) in two patients in the AC group. Attention-deficit/hyperactivity, autism spectrum, psychotic, and bipolar disorders were excluded. HC group members had no current or past history of psychiatric disorder. All participants reported no history of head trauma, serious medical/neurological illness, or MRI contraindications. Prior to participation in the study, and after thorough description of the study to potential participants and their parents, written informed consent/assent was obtained. Two participants were excluded due to technical problems resulting from poor co-registration, one patient was excluded due to an incidental brain finding, and one patient was excluded due to excessive motion. As a result of these exclusions (two OCD, one AC, and one HC), a total of 93 participants were included in the subsequent analyses. The research protocols described in this paper were approved by the Institutional Review Board within the UMMS (IRB numbers: HUM3920 and HUM00039079). Additional participant details are provided in Table 1.

**Table 1.** Participant characteristics

	Group			
	OCD	AC	HC	Statistic
*N*	23	26	44	
Age	14.00 (*3.84*)	13.38 (*3.20*)	14.55 (*3.88*)	*F* = 0.82, *p* = 0.445
Race	American Indian or Alaska Native: 1 Asian: 1 Black or African American: 1 Caucasian: 18 Multi-race: 1 Other: 1	American Indian or Alaska Native: N/A Asian: 1 Black or African American: 5 Caucasian: 17 Multi-race: 1 Other: 2	American Indian or Alaska Native: N/A Asian: 3 Black or African American: 4 Caucasian: 29 Multi-race: 6 Other: 2	N/A
MASC total^a^	57.11 (*18.61*) *n* = 18	57.12 (*21.08*) *n* = 26	30.53 (*14.76*) *n* = 38	*F* = 22.72, *p* < 0.001
Y-BOCS total	23.03 (*6.41*)	N/A	N/A	N/A
OCI-R total^a^	26.39 (*14.10*) *n* = 18	12.04 (*14.26*) *n* = 23	6.29 (*9.77*) *n* = 41	*F* = 17.11, *p* < 0.001
OCI-R checking^a^	3.06 (*3.04*) *n* *=* 18	1.73 (*2.66*) *n* = 22	0.86 (*1.93*) *n* = 37	*F* = 4.92, *p* = 0.010
OCI-R washing^a^	4.64 (*4.36*) *n* = 18	1.22 (*2.05*) *n* = 22	0.75 (*1.86*) *n* = 37	*F* = 13.16, *p* < 0.001
OCI-R ordering^a^	5.92 (*3.33*) *n* = 18	3.00 (*4.19*) *n* = 22	1.68 (*2.31*) *n* = 37	*F* *=* *10.77*, *p* < 0.001
OCI-R obsessing^a^	5.42 (*4.68*) *n* = 18	2.73 (*3.81*) *n* = 22	0.57 (*1.46*) *n* = 37	*F* = 14.21, *p* < 0.001
OCI-R hoarding^a^	3.89 (*3.32*) *n* = 18	2.32 (*2.66*) *n* = 22	2.32 (*2.45*) *n* = 37	*F* *=* 2.27, *p* = 0.111
OCI-R neutralizing^a^	3.47 (*3.76*) *n* = 18	1.22 (*3.05*) *n* = 22	0.41 (*1.32*) *n* = 37	*F* = 8.48, *p* < 0.001
FD	0.17 (*0.11*)	0.21 (*0.25*)	0.40 (*0.90*)	*F* = 1.31, *p* = 0.275
Comorbid tic, anxiety, and depressive^b^ disorders	Tics: 1 Anxiety: 8 Depressive: 4	Tics: 2 Anxiety: 11 Depressive: 8	Tics: 0 Anxiety: 0 Depressive: 0	N/A

Y-BOCS, Yale–Brown Obsessive Compulsive Scale; FD, framewise displacement.

a There were missing data for the Multidimensional Anxiety Scale for Children (MASC) Total and Obsessive Compulsive Inventory-Revised (OCI-R). Numbers of participants included in the calculations for each group are reported with *n* = *X* below the group means in the associated columns when there is missing data.

b Depressive disorders were dysthymia or depression not otherwise specified (NOS), with two patients in the anxiety group also meeting criteria for secondary major depressive disorder. The OCI-R subscale scores are scored from 0 to 12.

### Assessment of symptom severity and subtypes

OCD and anxiety symptoms were assessed in all subjects using the Obsessive Compulsive Inventory-Revised (OCI-R) and Multidimensional Anxiety Scale for Children (MASC). Symptom types were assessed with the OCI-R subscales. One-way analyses of variance were run to evaluate group differences in OCI-R and MASC scores. Symptom severity within the OCD patient group was also assessed using the appropriate child or adult version of the Yale–Brown Obsessive Compulsive Scale (C/Y-BOCS; Goodman et al., 1989).

### Imaging procedures/acquisition

Neuroimaging data were acquired on a 3.0T GE Signa scanner (General Electric, Milwaukee, Wisconsin) located at the University of Michigan Medical School. See online Supplement for details.

### Image preprocessing

Standard preprocessing of the imaging data was conducted using Data Processing & Analysis of Brain Imaging (DPABI; Yan, Wang, Zuo, and Zang, 2016) and Statistical Parametric Mapping (SPM12; https://www.fil.ion.ucl.ac.uk/spm/software/spm12/; Friston, 2003). See online Supplement for details.

### Functional connectivity analyses

We focused on OST, CON, and DMNs implicated in our previous study on OCD (Fitzgerald et al., 2011; Norman et al., 2019, 2021; Stern et al., 2012); whereas other networks may be involved in OCD pathology (de Vries et al., 2019; Stern et al., 2012), a concentration on these three networks allowed us to limit multiple comparisons. The OST striatal and thalamic regions of interest (ROIs) were derived from Di Martino et al. (2008) and Fitzgerald et al. (2011). All other ROIs, including the OFC ROI for the OST network, were selected from the Harvard–Oxford atlas distributed with FSL (http://www.fmrib.ox.ac.uk/fsl/), by placing a 5-mm spherical radius around the (approximate) center of each region as given by FSLview (https://fsl.fmrib.ox.ac.uk/fsl/fslwiki/FslView; Smith et al., 2004). ROIs for the DMN and CON were selected from previous categorizations of regions displaying patterns of correlated activity within each of these networks. The CON was composed of pMFC sub-regions in the dorsal anterior cingulate cortex (dACC), pre-supplementary motor area, and supplementary motor areas, as well as the frontal operculum, and anterior insula (Norman et al., 2019; Seeley et al., 2007). For the DMN, regions in precuneus, PCC, medial frontal pole, medial frontal gyrus, medial temporal lobe (MTL), and AG were selected (Yeo et al., 2011). Coordinates and corresponding Harvard–Oxford labels are provided in Table 2.

**Table 2.** Region labels and coordinates for CON, DMN, and OST ROIs

Region	Harvard–Oxford label	MNI: *x*, *y*, *z*	Network
Insula	Insular cortex	+/−40, 14, −8	CON
Frontal operculum	Frontal operculum cortex	+/−42, 22, 2	CON
Dorsal anterior cingulate cortex	Cingulate gyrus, anterior division	0, −4, 42	CON
Supplementary motor cortex	Juxtapositional lobule cortex (formerly supplementary motor cortex)	0, 0, 54	CON
Pre-supplementary cortex	Paracingulate gyrus	0, 34, 32	CON
Precuneus	Precuneus cortex	0, −64, 38	DMN
Posterior cingulate cortex	Cingulate gyrus, posterior division	0, −42, 36	DMN
Medial temporal lobe	Middle temporal gyrus, posterior division	+/−58, −8, −20	DMN
Angular gyrus	Angular gyrus	+/−48, −52, 38	DMN
Anterior medial prefrontal cortex	Frontal pole	0, 62, −2	DMN
Ventromedial prefrontal cortex	Frontal medial cortex	0, 42, −18	DMN
Caudate	–	+/−13, 15, 9	OST
Putamen	–	+/−28, 1, 3	OST
Thalamus	–	+/−8, −14, 8	OST
Orbitofrontal cortex	Frontal orbital cortex	+/−26, 36, −13	OST

MNI, Montreal Neurological Institute. CON, Cingulo-opercular network; DMN, Default mode network; OST, Orbitofrontal-striatal-thalamic circuit.

**Figure 1.**
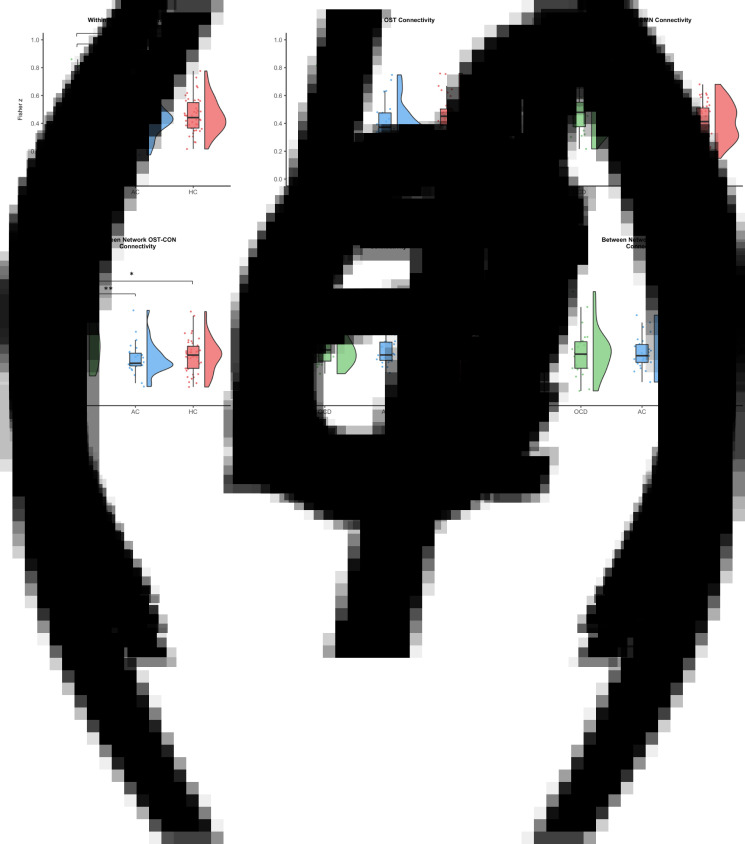
Raincloud plots displaying group differences in network connectivity within and between the CON, OST, and DMN. The OCD group is shown in green, the anxiety control group in blue, and the healthy control group in red. Each subfigure describes connectivity (a) within-CON, (b) within-OST, (c) within-DMN, (d) between OST–CON, (e) between OST–DMN, and (f) between DMN–CON. *Note*. Significance of post-hoc pairwise comparisons between groups is indicated with asterisks. *Refers to a significant difference between groups at *p* < 0.05. **Refers to a significant difference between groups at *p* < 0.01.

**Figure 2.**
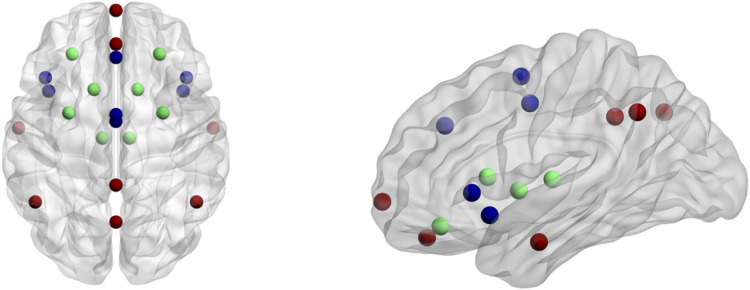
Bilateral ROIs of the OST (green), CON (blue), and DMN (red) presented on a glass brain. Region labels and coordinates are given in Table 2.

Time courses from all voxels within a 5-mm spherical radius around each of the ROI coordinates were averaged and then correlated with each other, thereby creating an ROI-to-ROI connectivity matrix for each subject containing all ROIs from the three networks (ROI coordinates are provided in Table 2). Fisher's *r*-to-*z* transformation was then applied to each cell of the resultant matrices. Within network connectivity was determined for *a priori* defined OST, CON, and DMNs by averaging the Fisher-*z*-transformed correlation coefficients for all intra-network correlations of each ROI, within each network, for each subject. Between network connectivity was calculated for each pairwise combination of networks (OST to CON, OST to DMN, and CON to DMN) by averaging all of the Fisher-*z*-transformed correlation coefficients of inter-network connections between each ROI of each of the two networks included in the network pair, for each subject. Separate one-way analysis of covariance (ANCOVA) models, including covariates of age and mean framewise displacement (FD), were used to compare the OCD, AC, and HC groups on measures of within network connectivity in each network, and network connectivity between each of the three networks. These tests were corrected for multiple comparisons using the Benjamini and Hochberg method (false-discovery rate; FDR; Benjamini and Hochberg, 1995). In order to interpret the group differences from the between-group ANCOVA, functional connectivity estimates were plotted in raincloud plots (Allen, Poggiali, Whitaker, Marshall, & Kievit, 2019), subjected to pairwise (OCD *v.* AC, OCD *v.* HC, AC *v.* HC group) post-hoc *t* tests, and corrected for multiple comparisons for three groups using the least significance difference method (Fisher, 1935) (Fig. 1). The ROIs for the three networks are depicted on a glass brain in Figure 2.

## Results

### Clinical characteristics

The average C/Y-BOCS score was 23.03 (6.41), indicating that OCD patients were experiencing symptoms of moderate severity. In the anxiety group, primary diagnoses were generalized anxiety disorder (*n* = 12), social anxiety disorder (*n* = 8), specific phobia (*n* = 3), panic disorder (*n* = 1), and separation anxiety disorder (*n* = 2); 10 patients had additional anxiety diagnoses (see Table 1). As expected, OCI-R scores were greater for the OCD group than that for the AC or HC groups. Scores on the MASC were greater in both the OCD and AC groups as compared to the HC group, and were similar between patient groups, reflecting the high rates of anxiety disorders typically observed in pediatric OCD (Geller et al., 2001; see Table 1). There were no group differences in mean age (*F*_(2,90)_ = 0.82, *p* = 0.45) or FD (*F*_(2,90)_ = 1.31, *p* = 0.28) and no significant correlation between FD and age (*r*(91) = −0.01, *p* = 0.90). See Table 1 for full descriptive and test statistics.

### Functional connectivity results

Functional connectivity within and between the OST, CON, and DMNs was assessed for group differences, controlling for age and mean FD. Main findings were group differences in connectivity within the CON (*F*_(2,88)_ = 4.91, *p* = 0.010, FDR-adjusted *p* = 0.045, *η*^2^_p_ = 0.10) and between the CON and OST networks (*F*_(2,88)_ = 4.38, *p* = 0.015, FDR-adjusted *p* = 0.045, *η*^2^_p_ = 0.09). Post-hoc tests revealed that within-network connectivity of the CON was greater in the OCD group (*M* = 0.54, S.E.= 0.027) than that in the AC (*M* = 0.43, S.E. = 0.025, *p* = 0.003) or HC groups (*M* = 0.46, S.E. = 0.020, *p* = 0.027). The AC group and HC group did not differ on the measure of within-CON connectivity (*p* = 0.238). Additionally, CON–OST between-network connectivity was greater in the OCD group (*M* = 0.40, S.E. = 0.027) than either the AC (*M* = 0.30, S.E.= 0.025, *p* = 0.004) or HC (*M* = 0.33,  S.E.= 0.020, *p* = 0.043) groups, which were not different from each other (*p* = 0.228). There were no further significant group differences in within- or between-network connectivity (all FDR-adjusted *p* > 0.214). Means and standard deviations for connectivity in and between the networks are reported in Table 3.

**Table 3.** Mean and standard deviations (S.D.) of within-network and between-network connectivity for the three groups

	OCD group		AC group		HC group		ANCOVA result
Network connectivity	Mean	S.D.	Mean	S.D.	Mean	S.D.	*p*FDR
Within-CON*	0.54	0.16	0.43	0.11	0.46	0.12	0.045
Within-DMN	0.47	0.13	0.42	0.13	0.41	0.14	0.426
Within-OST	0.49	0.17	0.42	0.14	0.45	0.12	0.214
CON–OST*	0.40	0.16	0.30	0.12	0.33	0.13	0.045
DMN–CON	0.15	0.18	0.15	0.12	0.12	0.12	0.627
OST–DMN	0.20	0.15	0.16	0.10	0.16	0.11	0.478

OCD, obsessive compulsive disorder; AC, anxiety control; HC, healthy control; CON, cingulo-opercular network; DMN, default mode network; OST, orbitofrontal–striatal–thalamic; *p*FDR, false-discovery rate corrected *p*-value. *Refers to cases where there was a significant group difference in connectivity.

### Secondary analyses

The C/Y-BOCS, collected only for the OCD group, was not significantly correlated with any of the six within- or between-connectivity metrics among OCD patients. In addition, the MASC was collected for most participants (*n* = 82), and was not significantly correlated with any functional connectivity metric in the three groups. The findings within-CON and between CON–OST remained significant after including an extra covariate for co-morbid depressive disorder. Additionally, the results held when participants ages >18 were excluded (see online Supplement).

## Discussion

The current study evaluated functional connectivity within and between the OST, CON, and DMN in patients with OCD as compared to AC and HC groups in female youth. Group differences were observed in two main domains. Specifically, a pattern of hyperconnectivity within the CON, and between the CON and OST networks, was found in the OCD group as compared to the other two groups at rest. Although we hypothesized that groups would differ in connectivity strength related to these networks, the pattern of differences between groups did not entirely match our expectations. For instance, among the three networks studied, we expected that only OST connectivity would distinguish the OCD from the anxiety disorder group; however, we found that connectivity differed within CON and between the CON and OST circuit for OCD youth relative to the AC youth. Within-CON hyperconnectivity was hypothesized for both OCD and AC groups, but was displayed only in the OCD group. Notably, the findings presented here indicate that there are connectivity profiles that are unique to female pediatric patients with OCD and not shared by age-matched patients with anxiety disorders.

### Within CON hyperconnectivity

The OCD group displayed significantly greater connectivity within the CON than the AC or HC groups. Evidence of task-based hyperactivity of the CON during error processing (Norman et al., 2019) is consistent with work implicating the CON broadly in functions of performance and error monitoring (Dosenbach, Fair, Cohen, Schlaggar, & Petersen, 2008; Neta et al., 2014; Sylvester et al., 2012), tonic alertness (Marek & Dosenbach, 2019), and electrophysiological studies demonstrating greater CON response to errors in patients with OCD (Gehring, Himle, & Nisenson, 2000; Hajcak, Franklin, Foa, & Simons, 2008; Hauser et al., 2017; Maltby, Tolin, Worhunsky, O'Keefe, & Kiehl, 2005). Although hyperconnectivity between these regions does not necessarily dictate task-related hyperactivity of these regions, a previous study found that when greater tonic alertness was required during a task, the regions of the CON became increasingly connected *and* active (Sadaghiani & D'Esposito, 2015). Therefore, it is possible that hyperconnectivity of the CON at rest in patients with OCD still reflects a hyperactive error monitoring mechanism even when no error is actively being made. This excessive error monitoring may translate to a pathological response in patients with OCD, for instance, as attempts are made to reduce error signals through repetitive behaviors or rituals (Hajcak et al., 2008; Pitman, 1987).

Greater error-related CON activity in OCD may reflect a pathologic (Hajcak et al., 2008) or a compensatory response (Fitzgerald et al., 2018; Moser, 2017) but, in either case, heightened levels of error-signaling within the CON could impact connectivity. Indeed, repeated cognitive activity has been found to increase connectivity of intrinsic networks (Lewis & Kim, 2009) such that, over time, high levels of error signaling in OCD could increase connections between CON nodes. To understand the possible influence of CON error-monitoring activity on CON connectivity in OCD, longitudinal research will need to examine the dynamic relationships between task-related and resting-state function of the CON over time. In theory, such research could especially benefit from the study of young patients, near illness onset, to capture any changes in connectivity resulting from a compensatory response to the symptoms themselves.

As with OCD, we predicted CON hyperconnectivity in the AC group based on literature suggesting hyperactive function across both disorders (Gillan, Fineberg, & Robbins, 2017). However, in contrast to OCD, we did not observe the hypothesized effect in AC. In order to distinguish the AC group from the HC group based on connectivity measures, different networks may need to be assessed. For instance, the frontoparietal network, ventral attention network, and connections between the amygdala and these two networks may be more relevant toward assessing AC-specific pathology (Sylvester et al., 2012). Indeed, meta-analysis suggests reduced anterior cingulate connectivity with amygdala in anxiety, rather than within the CON nodes studied here (Kolesar, Bilevicius, Wilson, & Kornelsen, 2019).

### OST–CON hyperconnectivity

An additional finding was that the OCD group, compared with the AC and HC groups, displayed hyperconnectivity between the OST circuit and the CON. Studies of the OST in OCD, relative to HCs, have found certain regions within this circuit to be anatomically enlarged (Hou et al., 2013), hyperactive and hyperconnected at rest (Harrison et al., 2009; Sakai et al., 2011), during symptom provocation (Breiter et al., 1996), and during habit-responding (Banca et al., 2015), but hypoactive during cognitive control and reward tasks in patients with OCD (Kang et al., 2013; Norman et al., 2019). Alterations in OST-mediated habit and motivation functions have been proposed to underlie an imbalance between competing OCD and goal-related behaviors in the disorder (Gillan et al., 2015). For instance, hyperconnectivity between the OST and CON may represent a greater influence of the habit system (localized to the OST circuit) over the brain circuitry responsible for salience detection and action selection (located in CON), which may manifest as compulsive actions (Chase et al., 2020; Gillan & Robbins, 2014; Graybiel & Rauch, 2000; Tomiyama et al., 2019) and cognitive inflexibility (Banca et al., 2015; Gu et al., 2008; Jung et al., 2017).

### OCD specificity

The observed differences in hyperconnectivity within the CON and between the CON and OST networks were unique to the OCD group; the AC group did not display heightened connectivity in and between these networks. These results suggest the potential of an OCD-specific mechanism that is observable in unmedicated, pediatric OCD patients. However, despite finding these differences in the current study, it is evident that OCD and non-OCD anxiety disorders share similar patterns of symptoms. Given these overlapping phenomena, it is also likely that symptoms of OCD and non-OCD anxiety disorders derive common underlying mechanisms (Fitzgerald, Schroder, & Marsh, 2020). Importantly, we have interpreted these current findings as one mechanism by which OCD may be distinct from non-OCD anxiety, with the understanding that there are likely many shared mechanisms as well, even specific to the functioning of the CON and other large-scale brain networks (Menon, 2011).

The distinct clinical presentation of OCD has led to its removal from the anxiety disorders chapter of *The Diagnostic and Statistical Manual of Mental Disorders* (5th ed.; DSM-5) into a separate section (APA, 2013; Morillo, Belloch, & García-Soriano, 2007), consistent with the notion of distinct underlying mechanisms. The current finding of greater within-CON and between CON–OST connectivity in youth with OCD, but not anxiety disorders, may reflect an excess of OST-driven habits and compulsive symptoms that are unique to OCD. However, anxiety symptoms experienced by OCD patients may also impact neural circuitry, and additional study is needed to disentangle mechanistic differences between OCD and non-OCD anxiety disorders. Specifically, patients with OCD and anxiety disorders should be studied throughout different phases of development and over time with progression of each symptom class to identify mechanisms that may distinguish these disorders.

### Limitations

Several limitations should be considered when interpreting the findings presented here. First, the sample used in the current study was composed of unmedicated, child, and adolescent female participants. The characteristics of this group allowed us to control for the potential confounding effects of gender (Zhang, Dougherty, Baum, White, & Michael, 2018) and extended illness as seen in adult patients (Andreescu, Sheu, Tudorascu, Walker, & Aizenstein, 2014) on intrinsic connectivity networks. However, it is important to consider that findings from the current study may not be generalizable to the broader OCD population. This is especially relevant because, although subtle gender differences in resting-state connectivity have been reported in typically developing youth, no studies to our knowledge have examined gender differences in fMRI studies of OCD. This would be a valuable question to assess in future research.

Additionally, the way in which brain networks are defined can impact connectivity findings (Fox & Greicius, 2010). Although the networks studied closely resemble their adult state during childhood and adolescence (Gilmore, Knickmeyer, & Gao, 2018), developmental changes in the relative strength of connections have been demonstrated (Fair et al., 2013; Grayson & Fair, 2017; Marek, Hwang, Foran, Hallquist, & Luna, 2015) and standard approaches for mapping connectivity in youth samples (e.g. child-specific brain atlases) are needed in the field and could inform future research.

Third, there was heterogeneity within the AC group. Although each participant in the OCD group had a primary diagnosis of OCD, the individuals in the AC group had primary diagnoses of different anxiety disorders. Finally, although the use of functional connectivity has greatly enhanced our understanding of large-scale brain differences across several psychiatric disorders (Brennan et al., 2019; Harrison et al., 2009; Woodward & Cascio, 2015), there is still much about this method that we do not understand. Specifically, without knowing the mechanism by which hyper- or hypo-connectivity within or between networks relates to behavior, interpretations of connectivity strength will continue to have limited clinical impact. Studies such as ours help to gain a better understanding of the OCD-specific abnormalities in functional connectivity, but future research will need to evaluate the mechanism by which a difference in connectivity relates to differences in behavior.

## Conclusion

Evaluation of functional connectivity within and between large-scale brain networks has offered an exciting mechanism for exploring neural dysfunction in OCD and anxiety disorders. Extending beyond the study of the anatomical OST circuit in OCD, this study provides new knowledge about potential brain network connectivity differences in the CON and interactions between the CON and the OST network in patients with OCD. Crucially, greater connectivity in and between the CON and OST circuits was found exclusively in young, unmedicated, female patients with OCD when directly compared to young, unmedicated patients with non-OCD anxiety. These findings are an important step toward identifying disorder- or symptom-specific markers for OCD.

## Supplementary material

For supplementary material accompanying this paper visit https://doi.org/10.1017/S0033291721003044. click here to view supplementary material
