# Supplementary material for: Disorder-specific cingulo-opercular network hyperconnectivity in pediatric OCD relative to pediatric anxiety
Source: Psychol Med. 2021 Aug 16;53(4):1468–78. doi: 10.1017/S0033291721003044 (PMC10009399; doi:10.1017/S0033291721003044)
Supplement: Supplementary file 1 [file S0033291721003044sup001.docx]

***Supplementary Information***

**Supplementary Methods**

*Imaging Procedures/Acquisition*. Foam padding around the head, in addition to participant instruction, were used to reduce head motion during scanning. For each scan session, a T1-weighted image was obtained to allow for alignment. Additionally, one 8-minute T2*-weighted functional reverse spiral sequence scan (repetition time 2000ms, echo time 30ms, FA = 90, field of view 20 cm, 40 slices, 3.0mm/slice, 64×64 matrix) was collected for each subject, for a total of 240 volumes. After the functional “resting-state” scan, a high resolution T1 scan (spoiled gradient recall, 1.5 mm slices, 0 skip) was collected and later used for anatomic normalization. During resting-state acquisition, participants were instructed to keep their eyes open, fixate on a white crosshair on a black background, and “allow the mind to wander.”

*Imaging Preprocessing.* The functional images from each participant were slice-time-corrected, to account for different acquisition times, and were spatially realigned to the first volume, to correct for head motion. ﻿Each individual’s high-resolution structural image was co-registered to the mean functional image. Five principal components derived from the segmented white matter and the cerebrospinal fluid images for each subject were regressed using the “CompCor” noise correction method (Behzadi, Restom, Liau, & Liu, 2007). Additionally, to minimize motion-related noise, the Friston 24-parameter model (Friston, Williams, Howard, Frackowiak, & Turner, 1996) was used to control for correlations resulting from in-scanner movement. Scrubbing was implemented for volumes with greater than 0.5mm of framewise-displacement (FD; Power, Barnes, Snyder, Schlaggar, & Peterson, 2012). A minimum of three minutes of data were needed in order to be included in the analyses. An average of 7.76 minutes (SD = .54; range = 2.5) of data were included for each person across the sample, although the minutes did not have to be consecutive. There was no significant difference in the minutes of data used across groups, *F*(2,90) = 2.48, *p* = .09. Time-series data were band-pass filtered (0.01-0.1 Hz) using the DARTEL toolbox (Ashburner, 2007) in SPM and functional data were normalized from individual native space to ﻿Montreal Neurological Institute (MNI) space (2mm voxel size) using the DARTEL toolbox (Ashburner, 2007) in SPM12. Finally, the data were spatially smoothed with a 6 mm^3^ full-width half-maximum Gaussian kernel.

**Supplementary Analyses**

To examine connectivity differences when patients 18-21 were excluded (to reduce the age range of the sample), post-hoc analyses were conducted. Group differences were significant only for the ANCOVAs evaluating within-CON (*F*(2, 76) = 5.08, *p* = .009, ﻿FDR adjusted *p* = .042) and between CON-OST (*F*(2, 76) = 4.51, *p* = .014, ﻿FDR adjusted *p* = .042) connectivity. For within-CON connectivity, the OCD group (M = .55, *SE* = .03) was significantly different from the AC group (M = .42, *SE* = .03, *p* = .004) and the HC group (M = .46, *SE* = .02, *p* = .037). Similarly, for between CON-OST connectivity, the OCD group (M = .43, *SE* = .03) was significantly different from the AC group (M = .30, *SE* = .03, *p* = .003) and the HC group (M = .33, *SE* = .02, *p* = .014). There were no other connectivity measures that displayed significant group differences in the sample of participants ages 8-17.

To assess the potential effects of comorbidity on our findings, we conducted independent samples *t*-tests to determine whether within-CON connectivity or between CON-OST connectivity differed between patients with comorbid diagnoses and patients without a comorbid diagnosis. For within-CON connectivity, there was no significant difference between patients with comorbidities (M = .48) and those without (M = .48), *t*(47) = -.19, *p* = .85. Similarly, for between CON-OST connectivity, there was no significant difference between patients with comorbidities (M = .33) and those without (M = .39), *t*(47) = -1.32, *p* = .19. Additionally, we re-ran the within-CON and between CON-OST ANCOVAs including comorbid depressive disorder as a covariate and the results remained the same as in the original analyses. For within-CON connectivity, there was a significant difference between groups (*F*(2, 92) = 4.56, *p* = .013). For between CON-OST connectivity, there was also a significant difference between groups (*F*(2, 92) = 3.80, *p* = .026).

**Supplementary References**

Ashburner, J. (2007). A fast diffeomorphic image registration algorithm. *Neuroimage*, *38*(1), 95-113. https://doi.org/10.1016/j.neuroimage.2007.07.007

Behzadi, Y., Restom, K., Liau, J., & Liu, T. T. (2007). A component based noise correction method (CompCor) for BOLD and perfusion based fMRI. *Neuroimage*, *37*(1), 90-101. https://doi.org/10.1016/j.neuroimage.2007.04.042

Friston, K. J., Williams, S., Howard, R., Frackowiak, R. S., & Turner, R. (1996). Movement‐related effects in fMRI time‐series. *Magnetic resonance in medicine*, *35*(3), 346-355. https://doi.org/10.1002/mrm.1910350312

Power, J. D., Barnes, K. A., Snyder, A. Z., Schlaggar, B. L., & Petersen, S. E. (2012). Spurious but systematic correlations in functional connectivity MRI networks arise from subject motion. NeuroImage, 59(3), 2142–2154. https://doi.org/10.1016/j.neuroimage.2011.10.018

**Supplementary Figure**

**Supplementary Figure 1.** Labeled ROIs of the cingulo-opercular (blue), orbito-striatal-thalamic (green) and default mode (red) networks presented on glass brains. **Full region labels and coordinates are given in Table 3.**
